# Rodent Malaria Erythrocyte Preference Assessment by an *Ex Vivo* Tropism Assay

**DOI:** 10.3389/fcimb.2021.680136

**Published:** 2021-07-12

**Authors:** Yew Wai Leong, Erica Qian Hui Lee, Laurent Rénia, Benoit Malleret

**Affiliations:** ^1^ Agency for Science, Technology and Research Infectious Diseases Laboratories (A*STAR ID Labs), Immunos, Biopolis, Singapore, Singapore; ^2^ Singapore Immunology Network, Agency for Science, Technology and Research (A*STAR), Immunos, Biopolis, Singapore, Singapore; ^3^ Department of Microbiology and Immunology, Immunology Translational Research Program, Yong Loo Lin School of Medicine, Immunology Program, Life Sciences Institute, National University of Singapore (NUS), Singapore, Singapore; ^4^ Lee Kong Chian School of Medicine, Nanyang Technological University, Singapore, Singapore

**Keywords:** erythrocyte tropism, reticulocyte, normocyte, erythrocyte invasion, rodent malaria, flow cytometry

## Abstract

Circulating red blood cells consist of young erythrocytes (early and late reticulocytes) and mature erythrocytes (normocytes). The human malaria parasites, *Plasmodium falciparum* and *P. vivax*, have a preference to invade reticulocytes during blood-stage infection. Rodent malaria parasites that also prefer reticulocytes could be useful tools to study human malaria reticulocyte invasion. However, previous tropism studies of rodent malaria are inconsistent from one another, making it difficult to compare cell preference of different parasite species and strains. *In vivo* measurements of cell tropism are also subjected to many confounding factors. Here we developed an *ex vivo* tropism assay for rodent malaria with highly purified fractions of murine reticulocytes and normocytes. We measured invasion into the different erythrocyte populations using flow cytometry and evaluated the tropism index of the parasite strains. We found that *P. berghei* ANKA displayed the strongest reticulocyte preference, followed by *P. yoelii* 17X1.1, whereas *P. chabaudi* AS and *P. vinckei* S67 showed mixed tropism. These preferences are intrinsic and were maintained at different reticulocyte and normocyte availabilities. Our study shed light on the true erythrocyte preference of the parasites and paves the way for future investigations on the receptor-ligand interactions mediating erythrocyte tropism.

## Introduction

Despite being enucleated, circulating erythrocytes are phenotypically diverse and range from young erythrocytes (which consist of early and late reticulocytes) to fully matured biconcave erythrocytes (normocytes). Early reticulocytes egress out of hematopoietic organs (primarily the bone marrow) into the peripheral circulation, where they only consist around 1-3% of total human erythrocytes (Chin-Yee et al., 1991). Human reticulocytes undergo extensive cellular changes as they complete their maturation into normocytes (Malleret et al., 2013), such as the loss of ribosomal RNA and the downregulation of many surface proteins (Wilson et al., 2016; Chu et al., 2018; Gautier et al., 2018).

The causative agent of malaria, *Plasmodium* spp., parasitizes on hepatocytes and erythrocytes of the vertebrate host. During blood-stage infection, parasite entry into erythrocytes is mediated by interactions between various parasite invasion ligands and their cognate erythrocyte surface receptors (Cowman et al., 2017). *P. vivax*, the most widespread human malaria species, has a stringent erythrocyte tropism and can only invade the most immature human reticulocytes (Malleret et al., 2015). It is thought that the reason behind this strict tropism is because *P. vivax* can only invade *via* receptors that are only present on reticulocytes, and indeed, two such reticulocyte-specific receptors, CD71 and CD98, were identified recently (Gruszczyk et al., 2018; Malleret et al., 2021). *P. falciparum*, the deadliest human malaria species, also has a preference for reticulocytes, but can invade normocytes too (Pasvol et al., 1980; Naidu et al., 2019). However, no reticulocyte-specific receptor for *P. falciparum* invasion has been identified so far (Cowman et al., 2017).

Due to obvious challenges of studying human malaria parasites, mouse models of malaria have always been an important part of malaria research (Zuzarte-Luis et al., 2014; De Niz and Heussler, 2018). There is some degree of conservation in the invasion pathways across different human and rodent malaria species, in terms of utilization of homologous parasite ligands (Iyer et al., 2007; Gunalan et al., 2013) and erythrocyte receptors (Swardson-Olver et al., 2002). This makes reticulocyte-preferring rodent malaria viable models to study human malaria reticulocyte invasion.

Historically, the erythrocyte tropism of rodent malaria parasites was studied by microscopy on blood samples of infected animals. From these studies, it is generally accepted that *P. berghei* ANKA and non-lethal strains of *P. yoelii* have a preference for reticulocytes (Fahey and Spitalny, 1984; Deharo et al., 1996; Cromer et al., 2006; Otsuki et al., 2009), whereas *P. vinckei* has a tendency to invade normocytes (Viens et al., 1971; Vigário et al., 2001). Another rodent malaria parasite, *P. chabaudi*, was reported to have a mixed tropism and equally prefers both reticulocytes and normocytes (Jarra and Brown, 1989). These studies, however, differ from one another in their ways of measuring erythrocyte preference, making tropism comparisons between different parasite species difficult. Microscopic evaluations are also subjective, and this problem is compounded by the difficulty of differentiating late-stage parasites in reticulocytes from those in normocytes. More recent tropism studies used flow cytometry to distinguish erythrocyte types by their levels of CD71 expression, but these are constrained to only certain species, like *P. berghei* (Dertinger et al., 2000; Thakre et al., 2018).

More importantly, *in vivo* tropism measurements have limitations in determining the true erythrocyte preference. First and foremost, blood samples of infected animals are only a ‘timepoint snapshot’ of erythrocyte types and do not represent the erythrocyte stage during the point of merozoite invasion. This problem is exacerbated by the potential acceleration of reticulocyte maturation after invasion, as seen in *P. vivax* (Malleret et al., 2015). Additionally, studies with *P. vivax* and *P. yoelii* have shown that infected reticulocytes are more prone to removal by the host’s cytotoxic CD8+ T-cells (Junqueira et al., 2018; Hojo-Souza et al., 2020), resulting in the underrepresentation of infected reticulocytes in circulation. Peripheral blood data are also made even more unreliable considering the extent of organ and deep vasculature sequestration of infected cells (Franke-Fayard et al., 2010; Claser et al., 2011), and the presence of extravascular parasite populations in hematopoietic organs (De Niz et al., 2018; Lee et al., 2018).

Here we comprehensively investigated the erythrocyte tropism of the four major rodent malaria species (*P. berghei*, *P. yoelii*, *P. chabaudi*, and *P. vinckei*) *in vivo* and *ex vivo*. To study their tropism *in vivo*, we used flow cytometry to clearly define infected reticulocytes and normocytes based on the expressions of reticulocyte-specific markers, CD71 and CD98. Realizing the need for a way to measure tropism without the influence of the above-mentioned *in vivo* confounders, we also developed an *ex vivo* flow cytometry-based tropism assay. Late-stage parasites were exposed to highly purified fractions of mouse reticulocytes and normocytes, and new invasions into each erythrocyte type were compared. The assay allows for a more accurate evaluation of the parasites’ intrinsic erythrocyte preference and cross-species comparisons.

## Results

### *In Vivo* Tropism of Rodent Malaria

To define mouse reticulocytes and normocytes *in vivo*, we utilized CD71 and CD98 as reticulocyte-specific markers. Circulating erythrocytes in naïve mice can be divided into three main populations: CD71+ CD98+, CD71+ CD98-, and CD71- CD98- erythrocytes. Based on their RNA content measured by thiazole orange staining, CD71+ CD98+ cells are the early reticulocytes, CD71+ CD98- cells are late reticulocytes, and CD71- CD98- cells are mainly normocytes (**Supplementary Figures S1A, B**). Interestingly, in malaria-infected mice, there also exists a population of CD71- CD98+ erythrocytes (**Supplementary Figure S1C**), whose role and relevance in the infection is still under investigation. For simplicity and to have a clear distinction between reticulocytes and normocytes, here we only focus on the CD71+ CD98+ reticulocytes and CD71- CD98- normocytes to measure erythrocyte tropism. Using this definition of erythrocyte types, we compared the erythrocyte tropism of the four rodent malaria species. These parasites strains have different parasitemia profiles in C57BL/6 mice (**Supplementary Figure S2**), so we compared erythrocyte tropism at two timepoints before the peak of infection for each strain. For the lethal strains, *P. berghei* ANKA (PbA) and *P. vinckei vinckei* S67 (PvvS67), we measured tropism before the mice started to succumb to the infection. For the self-resolving strains, *P. yoelii* 17X1.1 (Py1.1) and *P. chabaudi chabaudi* AS (PccAS), we measured tropism before the peak of parasitemia.

**Figure 1:**
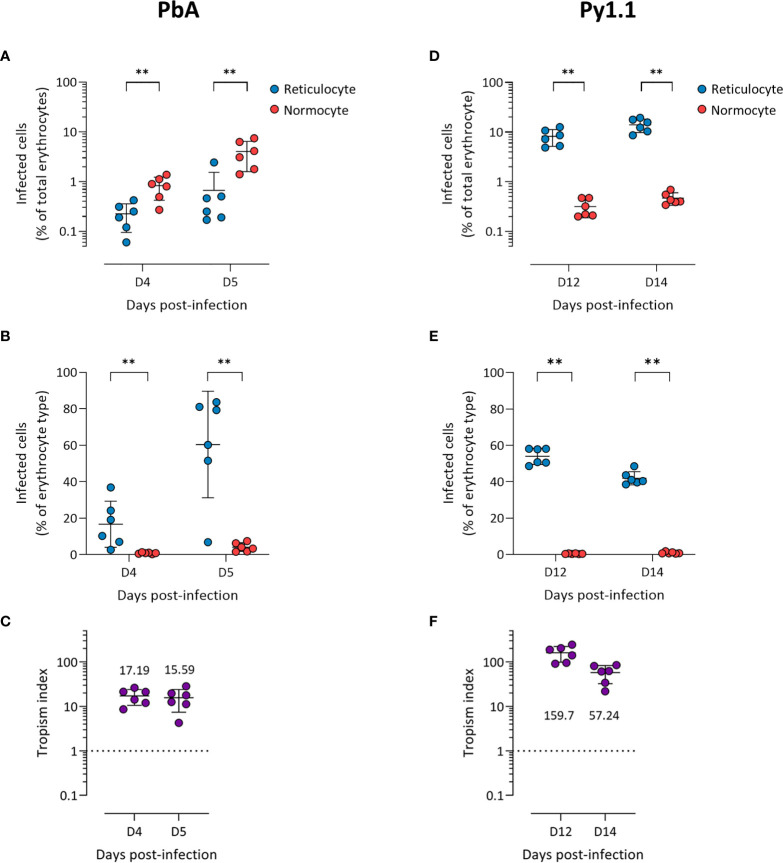
*P. berghei* ANKA **(A–C)** and *P. yoelii* 17X1.1 **(D–F)** prefer to invade reticulocytes *in vivo*. Flow cytometry data of peripheral blood taken from infected mice before the peak of infection (two days shown). Reticulocytes (blue circles) and normocytes (red circles) are defined as CD71+ CD98+ cells and CD71- CD98- cells, respectively. Infected cells are Hoechst-positive. **(A, D)** Data shown are infected reticulocytes and infected normocytes, as a percentage of total erythrocytes. **(B, E)** Data normalized to the relative frequencies of reticulocytes and normocytes, by expressing infected reticulocytes and normocytes as a percentage of total reticulocytes and total normocytes, respectively. **(C, F)** The *in vivo* tropism index is calculated as the ratio of percentage of infected reticulocytes (out of total reticulocytes) to percentage of infected normocytes (out of total normocytes). The mean tropism indices (*n* = 6 mice) are shown. Dotted lines at tropism index = 1 represent no erythrocyte preference. All error bars are standard deviations. **p < 0.01.

Before the peak of PbA infection, there were more infected normocytes than infected reticulocytes in circulation (**Figure 1A**), which could imply that PbA has a normocyte preference. However, normocytes were far more abundant than reticulocytes in circulation during this period (**Supplementary Figure S3**). This means that PbA merozoites were more likely to encounter and invade normocytes, therefore skewing the erythrocyte tropism. One way to normalize the different frequencies of reticulocytes and normocytes available is to compare the proportion of infected reticulocytes (out of total reticulocytes) with the proportion of infected normocytes (out of total normocytes). Using this approach, it is clear that PbA prefers to invade reticulocytes (**Figure 1B**). To further enable tropism comparisons across different strains, we also calculated the tropism index, which is a measure of how much more the parasite prefers reticulocytes over normocytes (see *Materials and Methods*). PbA had an *in vivo* tropism index of 15.6 (standard deviation, s.d. = 8.2) at 5 days post-infection (dpi), i.e. PbA prefers to invade reticulocytes 16 times more than normocytes (**Figure 1C**).

Py1.1-infected mice harbored more infected reticulocytes than infected normocytes before the peak of parasitemia (**Figure 1D**), and the proportion of infected reticulocytes were higher than the proportion of infected normocytes (**Figure 1E**). During this period, the number of circulating reticulocytes increased substantially due to the anemia-induced reticulocytosis (**Supplementary Figure S3**), therefore merozoites encounter reticulocytes and normocytes at almost similar frequencies. The tropism index of Py1.1 was more than 50 at 14 dpi (**Figure 1F**), indicating that its preference for reticulocytes is stronger than that of PbA.

**Figure 2:**
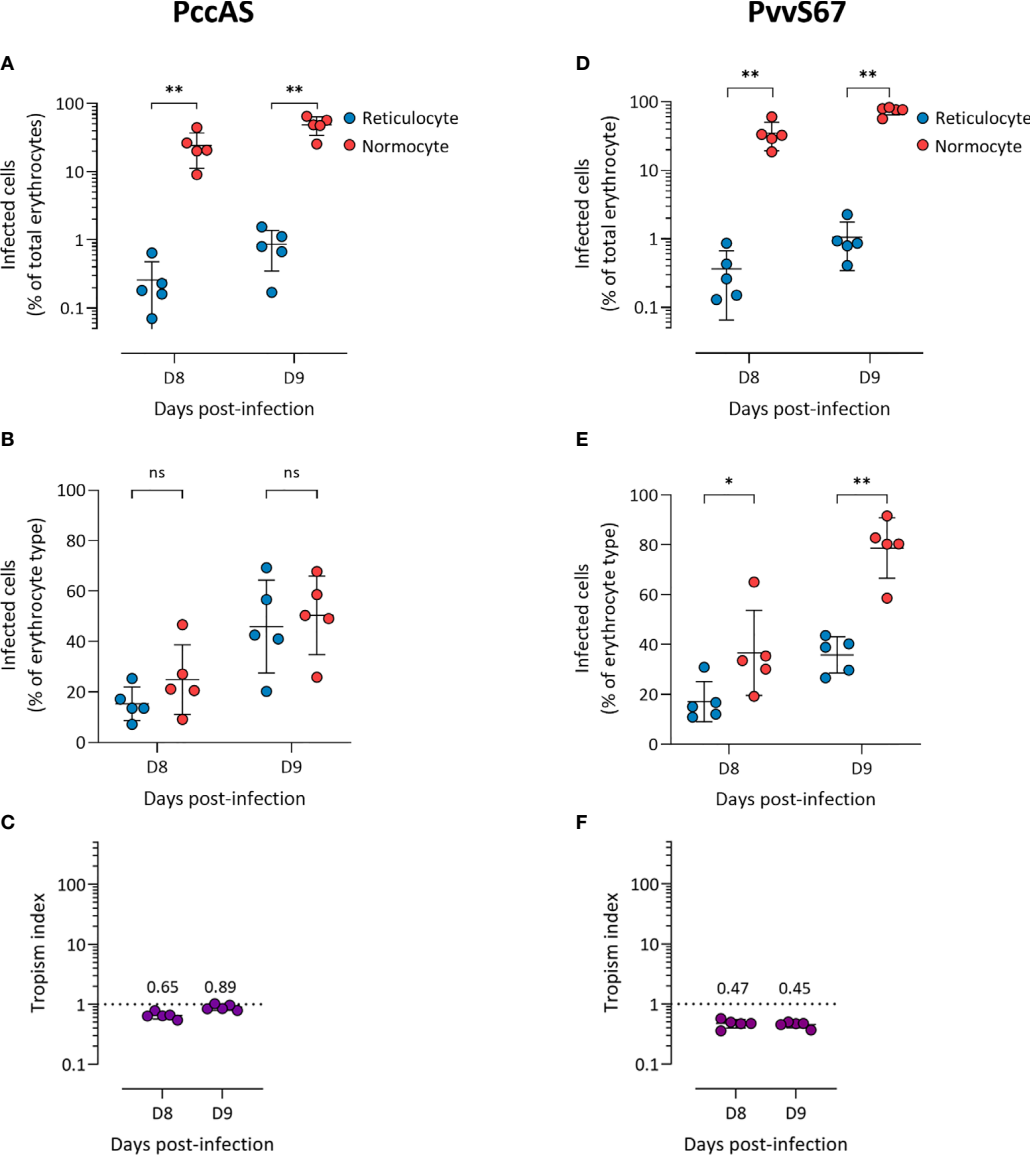
*P. chabaudi chabaudi* AS has no erythrocyte preference **(A–C)** and *P. vinckei vinckei* S67 prefers normocytes **(D–F)** *in vivo*. Flow cytometry data of peripheral blood taken from infected mice before the peak of infection (two days shown). Reticulocytes (blue circles) and normocytes (red circles) are defined as CD71+ CD98+ cells and CD71- CD98- cells, respectively. Infected cells are Hoechst-positive. **(A, D)** Data shown are infected reticulocytes and infected normocytes, as a percentage of total erythrocytes. **(B, E)** Data normalized to the relative frequencies of reticulocytes and normocytes, by expressing infected reticulocytes and normocytes as a percentage of total reticulocytes and total normocytes, respectively. **(C, F)** The *in vivo* tropism index is calculated as the ratio of percentage of infected reticulocytes (out of total reticulocytes) to percentage of infected normocytes (out of total normocytes). The mean tropism indices (*n* = 5 mice) are shown. Dotted lines at tropism index = 1 represent no erythrocyte preference. All error bars are standard deviations. ns, not significant; *p < 0.05; **p < 0.01.

We observed more PccAS-infected normocytes than infected reticulocytes in circulation (**Figure 2A**), but when the erythrocyte frequencies were normalized as previously described, PccAS did not show a significant preference for either erythrocyte type (**Figure 2B**). Its *in vivo* tropism index was 0.9 (s.d. = 0.1) at 9 dpi, which indicates a mixed tropism (**Figure 2C**). For PvvS67, both types of measures showed significantly higher invasion into normocytes than reticulocytes (**Figures 2D, E**). PvvS67’s tropism index is 0.5 (s.d. = 0.1) at 9 dpi, i.e. its preference for reticulocytes is half of its normocyte preference (**Figure 2F**). Altogether, our *in vivo* tropism studies verified that PbA and Py1.1 prefer reticulocytes, PccAS has no erythrocyte preference, and PvvS67 has a slight normocyte tropism. We were also able to compare the extent of reticulocyte preference across strains, and it is clear that Py1.1 has a higher reticulocyte preference than PbA *in vivo*.

### Development of an *Ex Vivo* Tropism Assay

**Figure 3:**
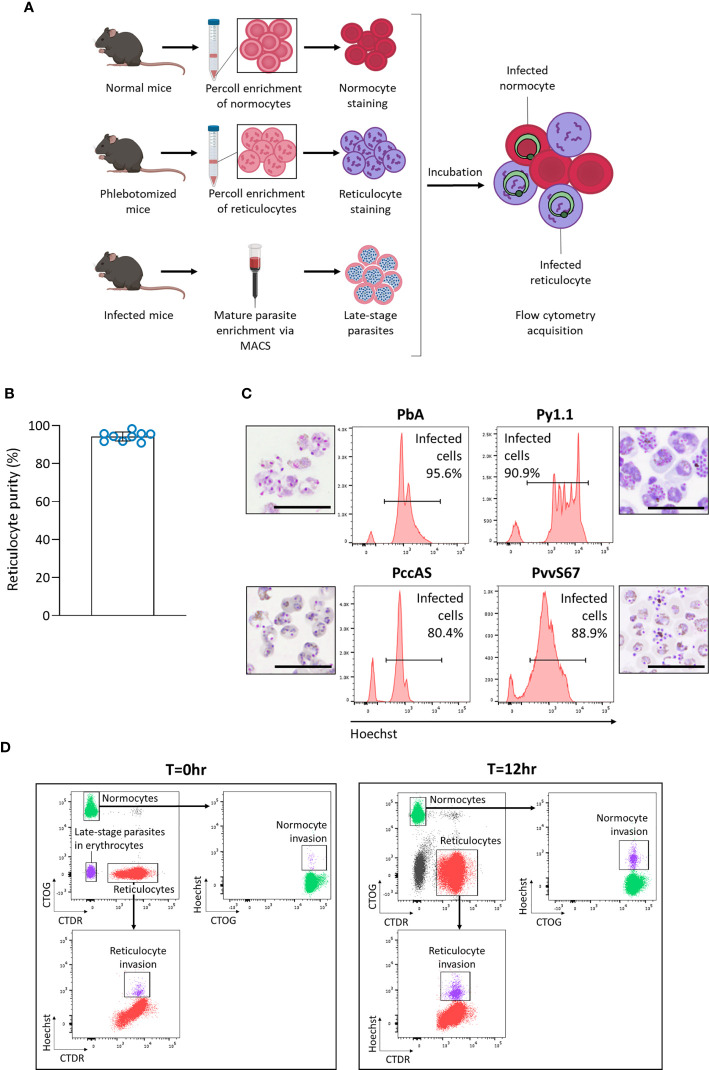
Development of an *ex vivo* tropism assay to measure erythrocyte preference of rodent malaria. **(A)** Schematic showing workflow of the assay. Normocytes are enriched by Percoll density centrifugation of blood taken from normal mice (untreated and uninfected). Reticulocytes are enriched similarly from blood taken from mice that underwent a phlebotomy routine. Enriched cells are then stained with different fluorescent dyes. Late-stage parasites are enriched from infected blood *via* magnetic-activated cell sorting (MACS). The cells are then incubated together for 12 hrs and measured by flow cytometry. Images were created on Biorender.com. **(B)** Reticulocyte purity (based on thiazole orange staining) after the two-step reticulocyte enrichment. Data from 9 independent experiments. Error bars represent standard deviation. **(C)** Late-stage parasite purity post-MACS for the four major rodent malaria strains. Histogram showing Hoechst levels and Giemsa-stained thin smears of enriched cells. Most of the enriched cells consist of late-stage parasites. Scale bar of microscopy images = 20 µm. PbA, *P. berghei* ANKA. Py1.1, *P. yoelii* 17X1.1. PccAS, *P. chabaudi chabaudi* AS. PvvS67, *P. vinckei vinckei* S67. **(D)** Flow cytometry gating strategy. At T=0hr, reticulocytes stained with CellTracker Deep Red (CTDR) are easily distinguishable from normocytes stained with CellTrace Oregon Green (CTOG). Late-stage parasites within erythrocytes are negative for both dyes. At T=12hr, new invasions into reticulocytes and normocytes can be detected by Hoechst.

We hypothesized that *in vivo* factors mentioned previously could skew tropism measurements, so we developed an *ex vivo* tropism assay to measure erythrocyte tropism in a controlled environment (**Figure 3A**). Late-stage parasites were incubated with enriched reticulocytes and normocytes, then new invasions into each cell type were measured by flow cytometry. To enrich mouse reticulocytes, mice were subjected to a phlebotomy routine to induce reticulocytosis and increase the number of circulating CD71+ CD98+ reticulocytes. Blood from these mice was further enriched for reticulocytes *via* Percoll density centrifugation. This two-step enrichment method consistently yielded ~ 95% reticulocyte purity (**Figure 3B**) and most of these reticulocytes were CD71+ CD98+ (**Supplementary Figure S4**). Importantly, this allowed the output of the assay to be directly compared with our *in vivo* data, in which we also defined reticulocytes as CD71+ CD98+ erythrocytes. Normocytes were similarly enriched from blood of normal (untreated and uninfected) mice and most of these cells were CD71- CD98- (**Supplementary Figure S4**).

The enriched reticulocytes and normocytes were then stained with different fluorescent cell dyes, CellTracker Deep Red (CTDR) and CellTrace Oregon Green (CTOG) respectively, so that they are distinguishable by flow cytometry (**Figure 3A**). To enrich late-stage parasites, blood from malaria-infected mice was subjected to magnet-activated cell sorting (MACS). MACS was able to efficiently enrich the hemozoin-containing late-stage parasites for all tested rodent malaria strains (**Figure 3C**). Finally, the parasites were incubated overnight with the target cells (stained reticulocytes and normocytes), and new invasions into each cell type were quantified by flow cytometry (**Figure 3D**). The CD71+ CD98+ reticulocytes did not mature considerably during incubation (**Supplementary Figure S5**), therefore we can be certain that newly-invaded cells in that population were reticulocytes during the point of merozoite invasion. Additionally, when the cell types were stained with the alternate fluorescent dyes, no significant difference in invasion was observed (**Supplementary Figure S6**), ruling out the possibility that the dyes were differentially affecting invasion into a particular cell type.

### *Ex Vivo* Tropism of Rodent Malaria at Equal Proportions of Reticulocytes and Normocytes

**Figure 4:**
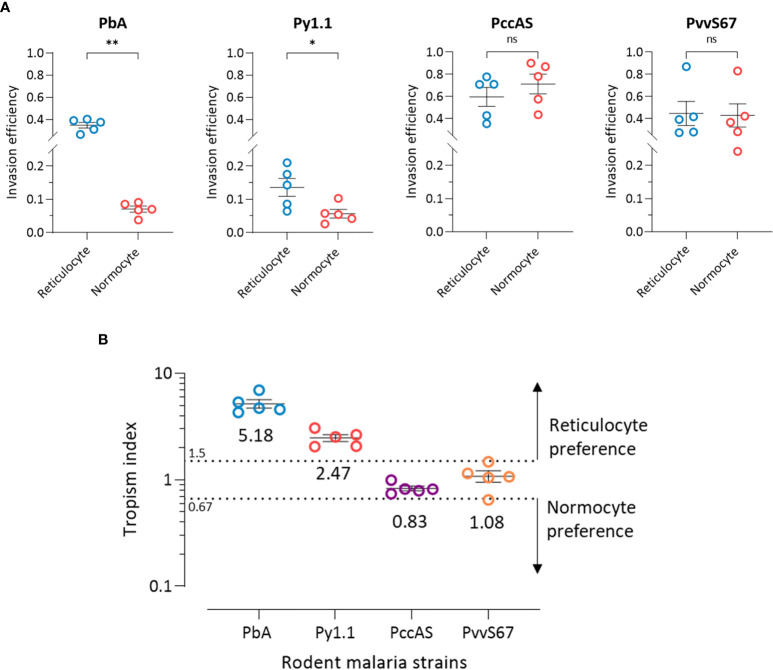
*Ex vivo* tropism of rodent malaria strains at equal reticulocyte:normocyte ratio. **(A)** Reticulocyte and normocytes invasion efficiencies (see *Materials and Methods* for calculation) were compared for all strains to determine erythrocyte preference. Data shown for each of the parasite strains are from 5 independent experiments. Error bars represent standard error of mean (SEM). ns, not significant; *p < 0.05; **p < 0.01. **(B)** Tropism indices are derived from the invasion efficiencies in **(A)**. Tropism index was calculated as the ratio of reticulocyte invasion efficiency to normocyte invasion efficiency. Dotted lines at tropism index = 0.67 and 1.5 represent the boundaries of mixed tropism, as determined by the spread of PccAS’s and PvvS67’s data. Tropism indices of > 1.5 indicate a preference for reticulocytes and tropism indices of < 0.67 indicate normocyte tropism. The mean tropism indices are shown, and error bars represent SEM. PbA, *P. berghei* ANKA. Py1.1, *P. yoelii* 17X1.1. PccAS, *P. chabaudi chabaudi* AS. PvvS67, *P. vinckei vinckei* S67.

The ratio of reticulocytes to normocytes in the tropism assay can be freely manipulated, therefore allowing us to measure the intrinsic erythrocyte preference of the parasite strains in an environment where reticulocytes and normocytes are equally available and not limited. At 50:50 reticulocyte:normocyte ratio, the reticulocyte invasion efficiencies of PbA and Py1.1 were significantly higher than normocyte invasion (**Figure 4A**). The assay also revealed no differences in reticulocyte and normocyte invasion for PccAS and PvvS67.

Considering the fact that PccAS and PvvS67 showed no erythrocyte preference *ex vivo*, we can define the baseline of reticulocyte preference at tropism index = 1.5 (1.5 times higher reticulocyte preference) and the baseline of normocyte preference at tropism index = 0.67 (1.5 times higher normocyte preference) (**Figure 4B**). With tropism indices of more than 1.5, both PbA and Py1.1 showed a marked tropism for reticulocytes. PbA was 5.2 (s.d. = 1.1) times more likely to invade a reticulocyte than a normocyte, and Py1.1 preferred reticulocytes 2.5 (s.d. = 0.4) times more than normocytes. In contrast to our *in vivo* data, PbA displayed a stronger preference for reticulocytes compared to Py1.1. Additionally, the tropism indices of PbA and Py1.1 were also much lower *ex vivo*. Overall, these results show that in an environment with equal availabilities of target erythrocytes, PbA and Py1.1 have an intrinsic preference for reticulocytes, whereas PccAS and PvvS67 can invade both erythrocyte types equally well.

### *Ex Vivo* Tropism of Rodent Malaria at Variable Proportions of Reticulocytes and Normocytes

Due to some disparity between *in vivo* and *ex vivo* observations, we next investigated whether target cell availability directly affects erythrocyte tropism. During the *in vivo* timepoints, normocytes represent a wide majority (>90%) of circulating erythrocytes for all parasite strains except Py1.1 (**Supplementary Figure S3**). Even though we tried to account for the vast difference in reticulocyte and normocyte availabilities by normalizing invasion to the respective cell frequencies, we cannot rule out that the excessive abundance of normocytes might be directly affecting invasion into a particular cell type. To simulate a wider range of erythrocyte frequencies, we varied the proportions of erythrocytes added to the tropism assay (10:90, 30:70, 50:50, 70:30, and 90:10 reticulocyte:normocyte ratio) and compared the invasion efficiencies.

**Figure 5:**
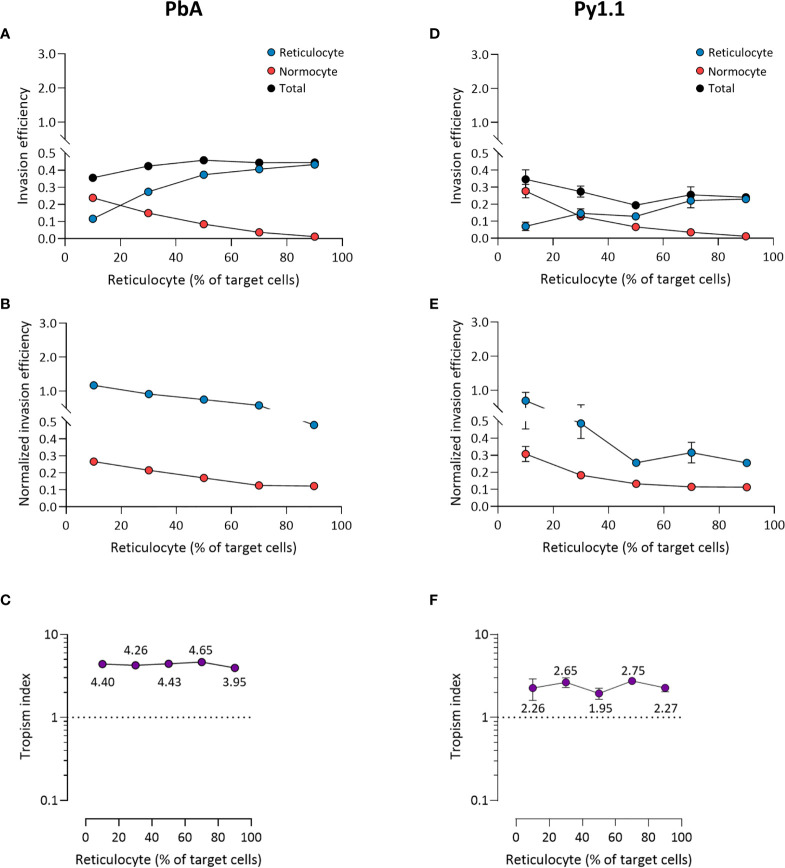
*Ex vivo* tropism of *P. berghei* ANKA **(A–C)** and *P. yoelii* 17X1.1 **(D–F)** at variable reticulocyte:normocyte ratios. **(A, D)** Reticulocyte (blue) and normocyte (red) invasion efficiencies (see *Materials and Methods* for calculation) were compared at five different erythrocyte ratios, 10:90, 30:70, 50:50, 70:30, 90:10 reticulocyte:normocyte. Total invasion efficiency (black) is the sum of reticulocyte and normocyte invasions. **(B, E)** Reticulocyte and normocyte invasion efficiencies were normalized to their respective population frequencies (see *Materials and Methods*). **(C, F)** Tropism indices were calculated from the ratio of normalized reticulocyte invasion efficiency to the normalized normocyte invasion efficiency. Mean tropism indices are also shown. Dotted lines at tropism index = 1 represent mixed tropism. All error bars represent standard deviation. *n* = 5 technical replicates, for each strain.

For PbA, there were more invaded reticulocytes than invaded normocytes at every ratio except when reticulocytes only constituted 10% of target cells (**Figure 5A**). However, when the invasion efficiencies were normalized to the corresponding cell population frequencies (see *Materials and Methods*), reticulocyte invasion was higher at every ratio (**Figure 5B**). This indicates that the parasite’s reticulocyte preference is intrinsic and unaffected by the surrounding erythrocyte composition. Reflecting this, PbA’s tropism index was stable across the different ratios (**Figure 5C**). Additionally, this shows that the tropism index is a reliable indicator of a parasite’s intrinsic erythrocyte preference. The results for Py1.1 are mostly similar to PbA, in which there were more infected normocytes only at the minimum reticulocyte frequency (**Figure 5D**). This superficial normocyte tropism was abolished once the relative erythrocyte frequencies were accounted for (**Figure 5E**). The tropism index of Py1.1 also remained constant at around 2 (**Figure 5F**).

**Figure 6:**
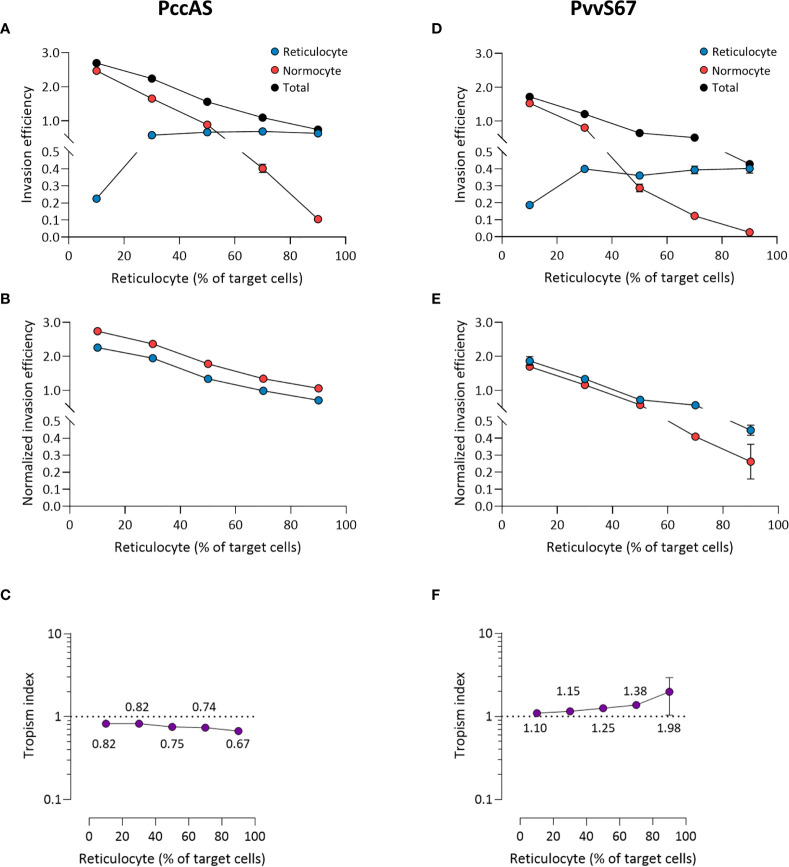
*Ex vivo* tropism of *P. chabaudi chabaudi* AS **(A–C)** and *P. vinckei vinckei* S67 **(D–F)** at variable reticulocyte:normocyte ratios. **(A, D)** Reticulocyte (blue) and normocyte (red) invasion efficiencies (see *Materials and Methods* for calculation) were compared at five different erythrocyte ratios, 10:90, 30:70, 50:50, 70:30, 90:10 reticulocyte:normocyte. Total invasion efficiency (black) is the sum of reticulocyte and normocyte invasions. **(B, E)** Reticulocyte and normocyte invasion efficiencies were normalized to their respective population frequencies (see *Materials and Methods*). **(C, F)** Tropism indices were calculated from the ratio of normalized reticulocyte invasion efficiency to the normalized normocyte invasion efficiency. Mean tropism indices are also shown. Dotted lines at tropism index = 1 represent mixed tropism. All error bars represent standard deviation. *n* = 5 technical replicates, for each strain.

The mixed tropism of PccAS was clearly seen at all erythrocyte ratios; an increase in reticulocyte invasion was accompanied by a decrease in normocyte invasion as the reticulocyte frequency increased, and the two invasion lines intersected around the 50:50 ratio (**Figure 6A**). Interestingly, the total invasion (sum of reticulocyte and normocyte invasions) decreased at higher reticulocyte frequencies, indicating that PccAS does not invade as well (regardless of target cell type) in a reticulocyte-rich environment. The mixed tropism of PccAS was stable at different ratios, as shown by the minimal difference between normalized invasion efficiencies (**Figure 6B**), and as shown by the tropism index (**Figure 6C**). PvvS67 also displayed similar trends to PccAS: the intersection of invasion lines near the 50:50 ratio (**Figure 6D**), the normalized invasion of both cell types were similar to one another at most ratios (**Figure 6E**), and the tropism index was mostly unchanged (**Figure 6F**). The total invasion of PvvS67 also decreased drastically at high reticulocyte numbers (**Figure 6D**). This was not seen in the reticulocyte-preferring strains, PbA (**Figure 5A**) and Py1.1 (**Figure 5D**). Taken together, these findings support the idea that erythrocyte preference of rodent malaria is intrinsic in nature and unaffected by the availability of target erythrocytes.

## Discussion

The surface proteome of reticulocytes and normocytes are remarkably different due to protein downregulation during reticulocyte maturation in humans (Wilson et al., 2016; Chu et al., 2018; Gautier et al., 2018) and mice (Gautier et al., 2020). Considering the fact that erythrocytic invasion by malaria parasites is mediated by erythrocyte receptor-parasite ligand interactions, the erythrocyte tropism of the parasites could hint at the key receptors necessary for invasion. However, when we attempted to classify rodent malaria species based on their erythrocyte tropism, we realized that previous tropism studies are inconsistent from one another in terms of measuring and evaluating tropism. This made cross-species comparisons difficult and there was no way to estimate their relative preferences for reticulocytes and normocytes.

Therefore, using flow cytometry to objectively define the erythrocyte populations, we performed comprehensive *in vivo* tropism measurements for the major rodent malaria strains. Our results broadly agree with previous observations: PbA and Py1.1 have a strong reticulocyte preference (Fahey and Spitalny, 1984; Deharo et al., 1996; Cromer et al., 2006; Otsuki et al., 2009; Martín-Jaular et al., 2013; Thakre et al., 2018), PccAS has a mixed tropism (Jarra and Brown, 1989), and PvvS67 has a slight normocyte tropism (Viens et al., 1971; Vigário et al., 2001). An important distinction between the present study and those before is that we only strictly compared normocytes with the earliest stages of reticulocytes (which still retain many surface proteins). This keeps the two erythrocyte populations well-defined and homogenous, allowing for more accurate measurements. The differential expression of surface proteins by the two populations also paves way to future mechanistic studies investigating the receptors mediating tropism. More importantly, our approach is able to rank the different rodent malaria strains based on their erythrocyte preference *in vivo*: Py1.1 (strongest reticulocyte preference) > PbA > PccAS > PvvS67 (strongest normocyte preference).

It is important to realize that the mere comparison of proportion of infected reticulocytes with the proportion of infected normocytes is not adequate in determining the true erythrocyte preference *in vivo*. Cromer et al. (2006) also realized this, and they attributed the problem to the dynamics of the infection; the changing rates of reticulocyte production and erythrocyte destruction could affect *in vivo* tropism measurements. They developed a mathematical model to account for this and concluded that *P. berghei* prefers reticulocytes ~150 times over normocytes.

Here we circumvented the problems of infection dynamics and other potential *in vivo* confounding factors (like parasite sequestration and preferential removal of infected reticulocytes) by developing an *ex vivo* tropism assay. In an environment where reticulocytes and normocytes were equally available, we observed that PbA and Py1.1 prefer reticulocytes (5 and 2.5 times more than normocytes, respectively), whereas PccAS and PvvS7 showed no inclination for either erythrocyte population. The most striking observation from this is that PbA’s and Py1.1’s reticulocyte preference *ex vivo* was much lower than our *in vivo* estimates (and the estimates in Cromer et al., 2006). We postulate that this discrepancy is due to the parasite’s capability to invade cells and replicate within hematopoietic sites of the host (e.g. bone marrow, spleen and liver), as supported by a growing number of evidence [reviewed in (Venugopal et al., 2020)] for *P. falciparum* (Joice et al., 2014), *P. vivax* (Obaldia et al., 2018), and *P. berghei* (De Niz et al., 2018; Lee et al., 2018). *P. berghei* was shown to parasitize early reticulocytes in the extravascular compartments of the bone marrow and spleen (De Niz et al., 2018; Lee et al., 2018). *P. yoelii* was also found in the bone marrow of infected mice (Imai et al., 2013). If a significant portion of invasion takes place in these reticulocyte-rich hematopoietic niches before the infected cells enter the bloodstream, then this would artificially inflate the reticulocyte preference of our peripheral blood data.

The role of the hematopoietic niche as a major developmental site for blood-stage malaria also raises an intriguing link with erythrocyte tropism. Do reticulocyte-preferring strains develop in hematopoietic organs due to the abundance of reticulocytes there, or is the hematopoietic environment inherently beneficial for parasite development and as an indirect consequence, the parasites have adapted to invading the cells present there (which happens to be reticulocytes)? On the contrary, we also observed that PccAS and PvvS67, which do not have the propensity to invade reticulocytes, have reduced total invasion efficiencies in a reticulocyte-rich environment. It is likely that these strains primarily invade in the peripheral circulation, where reticulocytes are less abundant, as opposed to PbA and Py1.1.

Notably, none of the strains tested here showed a preference for normocytes, which is surprising considering that normocytes are easily accessible and constitute the bulk of circulating erythrocytes. This could be due to the possibility that many of the receptors used for normocyte invasion are also found in similar abundance (if not more) on reticulocytes. Another possible explanation is that it is directly beneficial for the parasite to invade reticulocytes due to the relatively (compared to normocytes) metabolic-rich environment of the cells. Reticulocytes still retain some metabolic pathways that can be capitalized by the parasites (Srivastava et al., 2015). Reticulocyte invasion could also be a parasite evasion strategy; reticulocytes express higher levels of the ‘do not eat me’ signal, CD47, allowing infected reticulocytes to escape phagocyte clearance (Banerjee et al., 2015).

To sum up our *ex vivo* findings, PbA showed the strongest preference for reticulocytes, followed by Py1.1, whereas both PccAS and PvvS67 showed mixed tropism. Since the assay is devoid of external factors that could affect tropism, we believe that PbA’s and Py1.1’s strong reticulocyte preference is indicative of the erythrocyte receptors that are essential for their invasion. These unidentified receptors could be more abundant on the surface of reticulocytes than normocytes, allowing a higher probability of successful invasion into reticulocytes. Another possibility is that these receptors are only found on reticulocytes, but the parasites are also able to utilize less-preferred receptors on normocytes. A wide range of host receptors have been identified for *P. falciparum* invasion (Cowman et al., 2017), but none of them explain its tropism (prefers reticulocytes but can also invade normocytes). We suggest that PbA and Py1.1 are good mouse models to study *P. falciparum* invasion, considering that they show similar tropism signatures. Even though none of the rodent malaria strains tested here (and by others) showed a strict tropism to reticulocytes like *P. vivax*, reticulocyte-preferring strains still remain potential models to identify new reticulocyte-specific receptors. Our work here hopefully encourages deeper investigations into determining the receptor-ligand interactions mediating tropism, and identifying interventions that inhibit these interactions, consequently halting the reinvasion of erythrocytes.

## Materials and Methods

### Mice

Male and female C57BL/6J mice (aged 5-9 weeks old) were used. For reticulocyte and normocyte enrichment, mice were age- and gender-matched. Mice were housed under specific pathogen-free (SPF) conditions in the A*STAR Biomedical Resource Centre (Biopolis, Singapore). All animal procedures were approved by the A*STAR Biomedical Resource Centre Institutional Animal Care and Use Committee (IACUC #181314) in accordance with guidelines set by the Animal & Veterinary Service (AVS) and National Advisory Committee for Laboratory Animal Research (NACLAR) of Singapore.

### Parasite Strains and Mice Infection

The rodent malaria strains used were *Plasmodium berghei* ANKA clone 15cy1 (PbA) (Janse et al., 2006), *P. yoelii* 17X clone 1.1 (Py1.1) (Weiss et al., 1989), *P. chabaudi chabaudi* clone AS (PccAS) (Cornelissen et al., 1985) and *P. vinckei vinckei* S67 (PvvS67) (Bafort, 1967). The origin of the different parasite strains was ascertained using a combination of 19 microsatellite probes described elsewhere (Li et al., 2007). Parasite stocks were made in Alsever’s buffer and stored in liquid nitrogen. Mice were infected intraperitoneally (i.p.) with 10^6^ infected erythrocytes from the parasite stocks.

### *In Vivo* Tropism Follow-Up *via* Flow Cytometry

1 µL tail blood of infected mice was collected into 100 µL phosphate-buffered saline (PBS). Samples were stained with Hoechst 33342 (at 8 µM; Sigma #B2261), rat IgG2b anti-mouse CD45-PE/Cy7 (at 2 µg/mL; BD #552848), rat IgG2a anti-mouse CD71-APC (at 2 µg/mL; Invitrogen #17-0711-82), and rat IgG2a anti-mouse CD98-PE (at 2 µg/mL; Invitrogen #12-0981-83) for 15-20 mins. Cells were washed with PBS and immediately acquired on the BD LSRII flow cytometer, with a minimum of 100,000 cells recorded. Data were analyzed using FlowJo software (version 10). Erythrocytes were gated as CD45- cells. Tropism index for *in vivo* experiments was calculated as the ratio of the proportion of CD71+ CD98+ reticulocytes that were infected, to the proportion of CD71- CD98- normocytes that were infected.

### Late-Stage Parasite Enrichment

Blood from infected mice was collected into heparinized parasite media (RPMI 1640 + 20% heat-inactivated fetal bovine serum (FBS) + 40 µg/mL gentamicin). Leukocytes were depleted by passing the blood suspension through non-woven fabric (NWF) filter twice (Tao et al., 2011), and washed with parasite media. Late-stage parasites of all rodent malaria strains were enriched *via* magnet-activated cell sorting (MACS). Infected blood was passed through an LD column (Miltenyi Biotec #130-042-901) attached to a magnet. Late-stage parasites, which were retained in the column, were flushed out with parasite media. The purity of late-stage parasites was assessed with Hoechst staining and Giemsa-stained thin smears. Among the late-stage parasites, trophozoites and schizonts represent the majority stages for all parasite strains (>95%) (**Supplementary Figure S7**). Although there were some uninfected erythrocytes present in the late-stage parasite-enriched fraction, these cells only make up <2% of total cells after the addition of target erythrocytes, and therefore unlikely to affect the invasion of target cells.

### Reticulocyte and Normocyte Enrichment

Mice were subjected to an adapted phlebotomy routine to induce reticulocytosis (Liu et al., 2010). On Days -4 and -2, isoflurane-anesthetized mice were bled 600 µL *via* the retro-orbital route and injected with 1 mL PBS i.p. to replace the loss of fluid. On Day 0, the mice were exsanguinated under anesthesia into heparinized parasite media. The reticulocyte-rich blood suspension was leukodepleted and washed as previously, then resuspended to 50% hematocrit with parasite media. To enrich reticulocytes by Percoll density centrifugation, 2 mL 45% isotonic Percoll was overlaid on 3 mL 70% isotonic Percoll, and 1 mL of the blood suspension was laid on the 45% layer. The blood was then centrifuged at 250 x *g* for 30 mins at room temperature. The reticulocyte band, which can be found at the interface of the two Percoll layers, was removed carefully and washed with parasite media. To enrich normocytes, normal mice were bled and the blood was processed similarly to above. After Percoll centrifugation, normocytes were collected from the pellet. The purity of enriched reticulocytes and normocytes were assessed by staining with thiazole orange (at 0.5 µg/mL; Sigma #390062), 1 µg/mL anti-mouse CD71-APC and 1 µg/mL anti-mouse CD98-PE for 15-20 mins.

### *Ex Vivo* Tropism Assay

Enriched reticulocytes and normocytes were resuspended to 1% hematocrit with PBS and stained with CellTracker Deep Red (at 2 µM; Invitrogen #C34565) and CellTrace Oregon Green (at 15 µM; Invitrogen #C34555), respectively. After 30 mins of staining at 37°C, warm FBS was added to stop the staining reaction. Cells were then washed twice with media, with another 10 mins incubation during the second wash. Stained reticulocytes and normocytes were mixed to the preferred ratios (10:90, 30:70, 50:50, 70:30, or 90:10) and resuspended in parasite media to 2% hematocrit. Enriched late-stage parasites were then added at around 5-10% final parasitemia and the cells were incubated at 37°C for 12 hours. At Time = 0 hr (i.e. after the addition of parasites), aliquots of samples were mixed with PBS and stained with 8 µM Hoechst for 15-20 mins. 300 µL PBS was then added to quench the reaction and the samples were immediately acquired. The staining and flow cytometry acquisition was repeated at Time = 12 hr. This 12-hour incubation allows sufficient time for the trophozoites and schizonts to develop and release merozoites, and yet not too long until a second round of invasion occurs [e.g. the asexual cycle of different *P. yoelii* strains vary between 18-24 hours (Killick-Kendrick and Warben, 1968; Gautret et al., 1994)] or for the target reticulocytes to mature into normocytes.

### Invasion Efficiency Calculation and Normalization

$$Invasion\;efficiency=\left[\frac{\left(T12h\;invasion-T0h\;invasion\right)}{T0h\;Late\;stage\;parasites}\right]\times 0.5$$

The equation above shows how invasion efficiencies of reticulocytes and normocytes were calculated for the 50:50 reticulocyte:normocyte ratio experiments. The values for ‘T0h reticulocyte invasion’, ‘T12h reticulocyte invasion’, ‘T0h normocyte invasion’, ‘T12h normocyte invasion’, and ‘T0h Late-stage parasites’ were directly taken from the flow cytometry gates (see **Figure 3D**). The resultant value (in squared brackets) is then multiplied by 0.5 because reticulocytes and normocytes each represent half of the target cell population. For the variable ratios experiments, instead of 0.5, the resultant value is multiplied accordingly. For example, to determine the reticulocyte invasion efficiency when reticulocytes consist 90% of target cells, the resultant value is multiplied by 0.9 instead.

To normalize the invasion efficiency to the corresponding cell type frequency, the invasion efficiency is divided by the cell frequency. For example, to get the normalized reticulocyte invasion efficiency when reticulocytes consist 90% of target cells, the reticulocyte invasion efficiency is divided by 0.9. Consequently, the normalized value is equivalent to the value in squared brackets.

### Statistical Analysis

Statistical analyses were performed on GraphPad Prism software (version 9). All data were analyzed non-parametrically using Mann-Whitney test, and *p*-values < 0.05 were considered statistically significant.

## Data Availability Statement

The raw data supporting the conclusions of this article will be made available by the authors, without undue reservation.

## Ethics Statement

The animal study was reviewed and approved by A*STAR Biomedical Resource Centre Institutional Animal Care and Use Committee (IACUC).

## Author Contributions

YL, LR, and BM conceptualized and designed the study. YL and EL performed the experiments. YL, EL, LR, and BM analyzed and interpreted the data. YL, LR, and BM prepared the manuscript. All authors contributed to the article and approved the submitted version.

## Funding

The project was supported by core funds given by the Agency for Science, Technology and Research (A*STAR) to the Singapore Immunology Network (SIgN) (to LR and BM) and A*STAR ID Labs (to LR); and by the National University Health System (NUHS) Start-up grant (NUHSRO/2018/006/SU/01) (to BM). YL is funded by the Singapore International Graduate Award (SINGA) by A*STAR. The funding sources had no role in the study design; collection, analysis and interpretation of data; in the writing of the report; and in the decision to submit the paper for publication.

## Conflict of Interest

The authors declare that the research was conducted in the absence of any commercial or financial relationships that could be construed as a potential conflict of interest.
