# Supplementary material for: Rodent Malaria Erythrocyte Preference Assessment by an Ex Vivo Tropism Assay
Source: Front Cell Infect Microbiol. 2021 Jul 12;11:680136. doi: 10.3389/fcimb.2021.680136 (PMC8311856; doi:10.3389/fcimb.2021.680136)
Supplement: Supplementary file 1 [file DataSheet_1.docx]

Supplementary Material

**
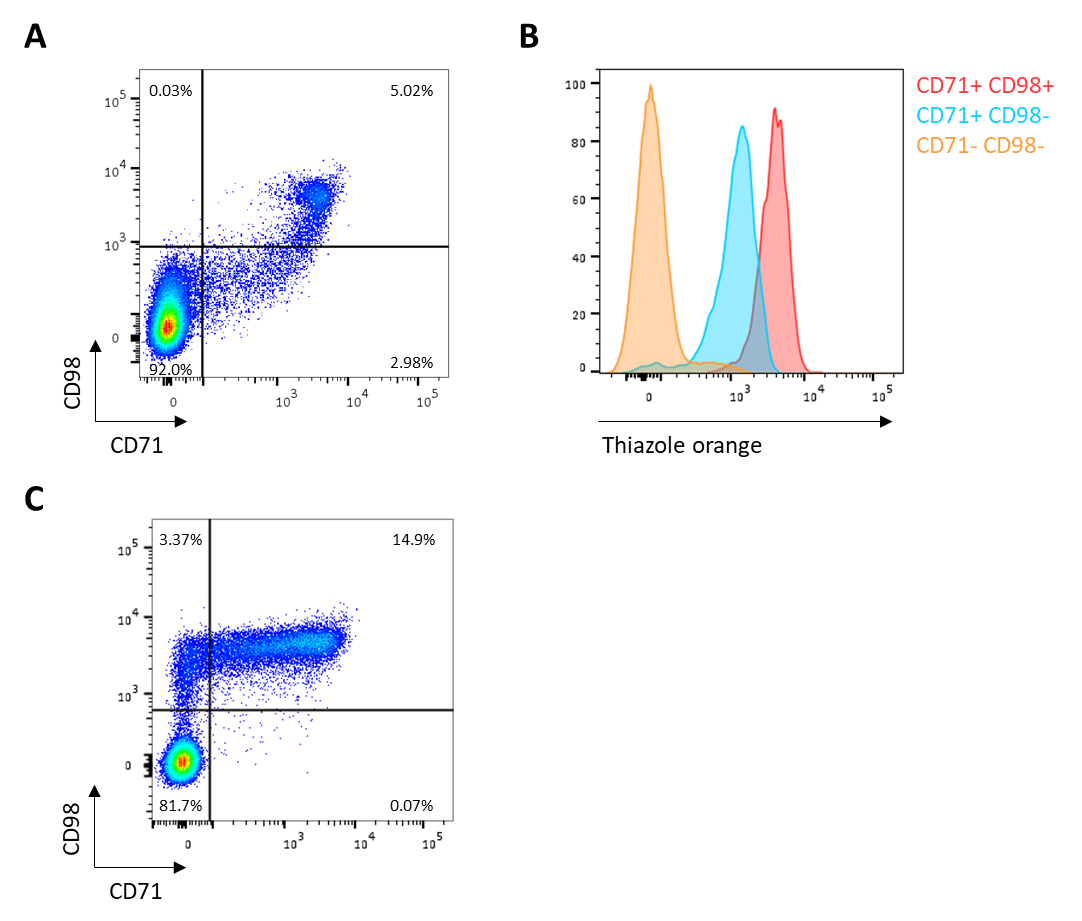
**

**Supplementary Figure S1. (A)** Flow cytometry dotplot showing CD71 and CD98 expression of circulating erythrocytes (gated from CD45- cells). There were three main populations: CD71+ CD98+, CD71+ CD98-, and CD71- CD98- cells. **(B)** Histogram showing RNA content, based on thiazole orange staining, of the three erythrocyte populations. Younger erythrocytes contain more RNA. **(C)** Representative dotplot of circulating erythrocytes from *P. yoelii* 17X1.1-infected mouse (12 dpi), showing the appearance of CD71- CD98+ erythrocytes. This erythrocyte population is also observed in infections of the other parasite strains mentioned in this study.


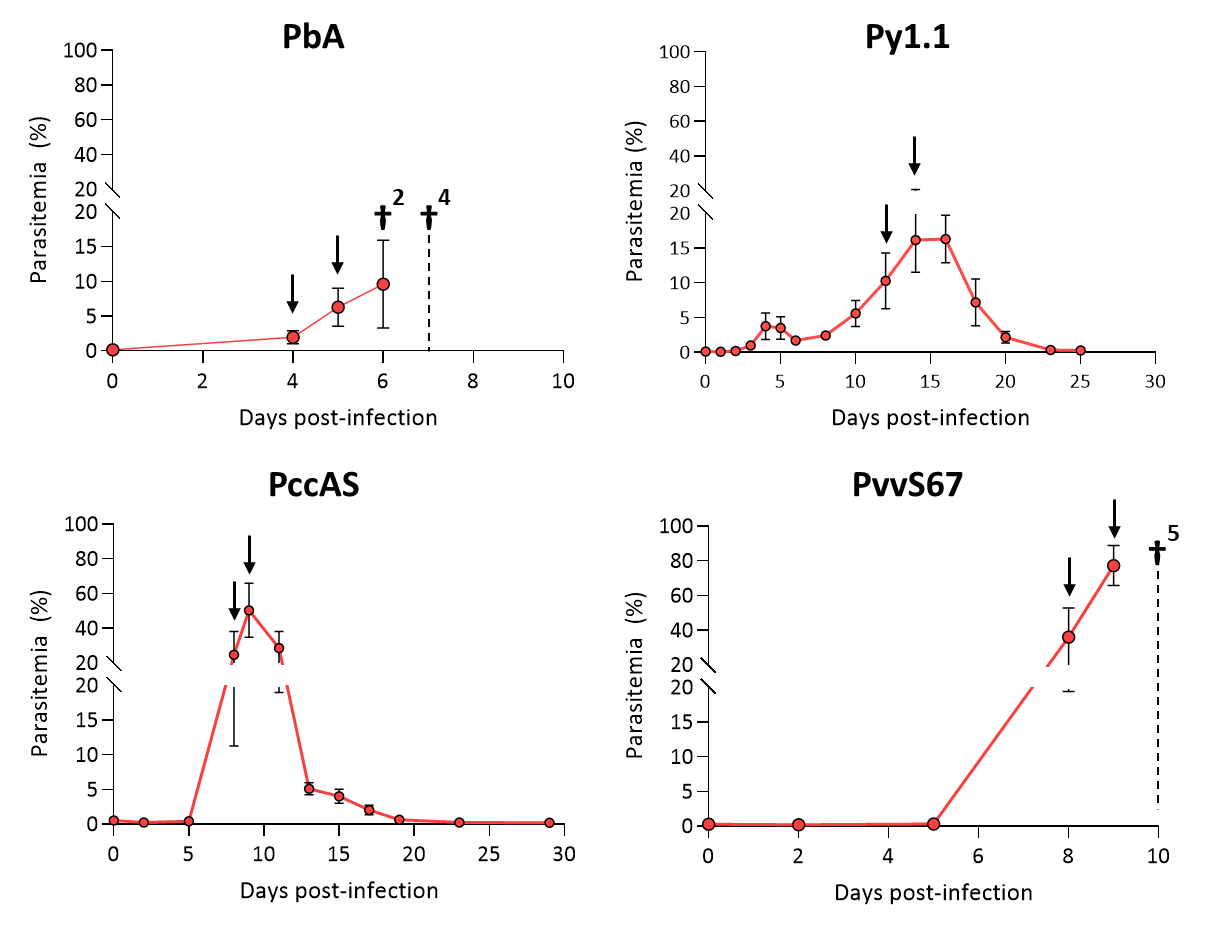


**Supplementary Figure S2.** Parasitemia profile of rodent malaria strains in C57BL/6 mice. Infected cells were identified by Hoechst staining. Crosses with superscripts represent number of mouse deaths on that particular day. Timepoints chosen to measure *in vivo* erythrocyte tropism are represented by arrows. For PbA (*n* = 6) and PvvS67 (*n* = 5), which cause lethal infections, timepoints before mouse deaths were chosen. For Py1.1 (*n* = 6) and PccAS (*n* = 5), which cause self-resolving infections, timepoints before the peak of parasitemia were chosen. Error bars represent standard deviations.


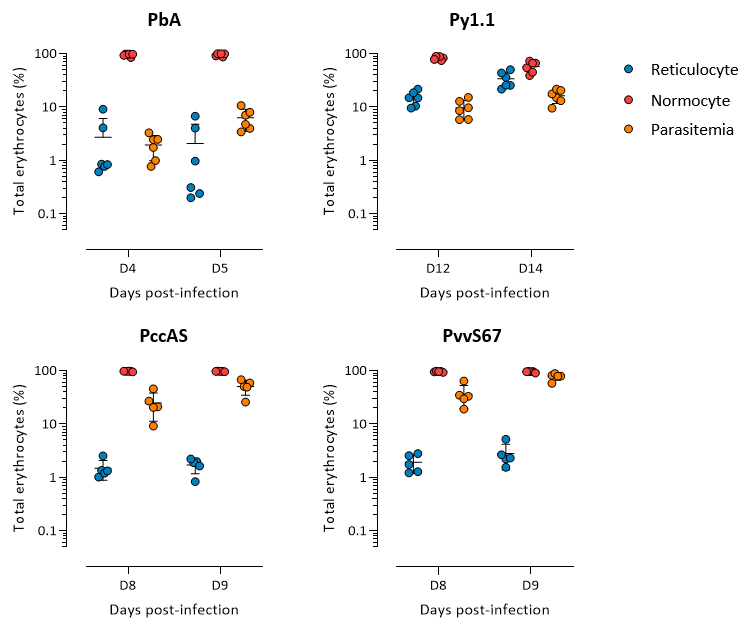


**Supplementary Figure S3**. Peripheral blood parasitemia and frequencies of erythrocyte types before the infection peak for the tested rodent malaria strains. Erythrocytes were gated as CD45- cells. Reticulocytes and normocytes were defined as CD71+ CD98+ and CD71- CD98- cells, respectively. Error bars represent standard deviation.


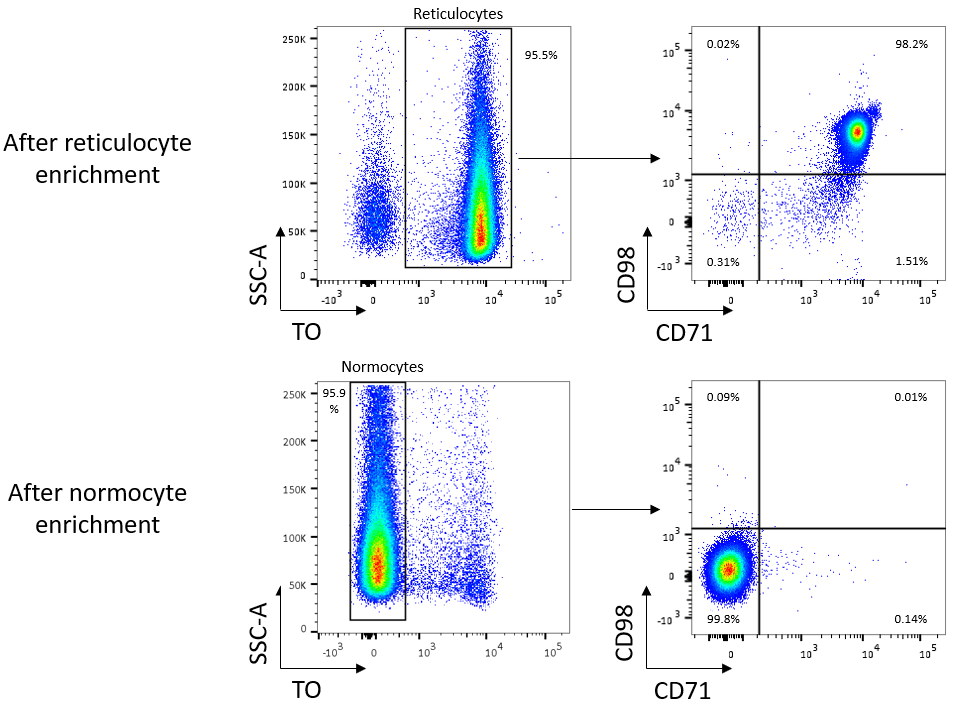


**Supplementary Figure S4.** Representative flow cytometry dotplots of enriched reticulocytes and normocytes after Percoll density centrifugation. Most enriched reticulocytes were CD71+ CD98+ and enriched normocytes were CD71- CD98-. TO, thiazole orange.


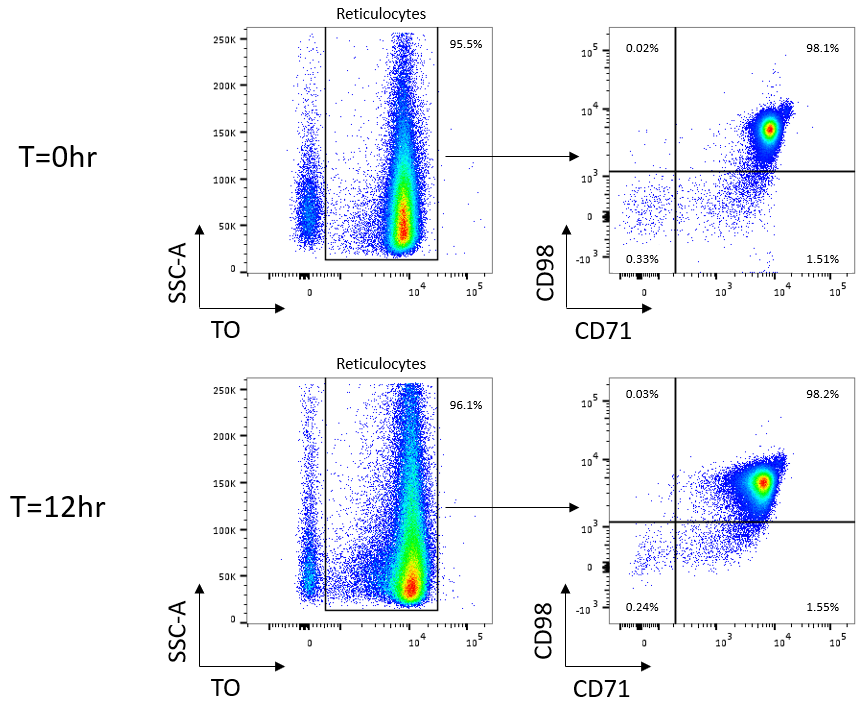


**Supplementary Figure S5.** Flow cytometry dotplots showing that enriched CD71+ CD98+ reticulocytes did not mature after 12 hrs of incubation. They also retained expression of CD71 and CD98. TO, thiazole orange.


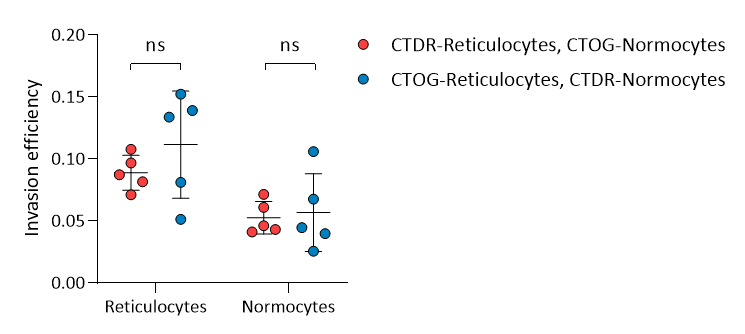


**Supplementary Figure S6.** Reticulocyte and normocyte invasion efficiencies were unchanged when the cells were stained with the alternate fluorescent dyes. Error bars represent standard deviations. CTDR, CellTracker Deep Red. CTOG, CellTrace Oregon Green. ns, not significant. Statistical test used was Mann-Whitney test.

**Supplementary Figure S7.** Composition of enriched late-stage parasites after magnet-activated cell sorting (MACS). Trophozoites and schizonts represent the majority stages for all rodent parasite strains tested. Error bars represent standard deviation. *n* = 3 independent experiments for each strain.
